# Pulmonary Artery Pseudoaneurysms Embolization: Bicentric Experience and Review of the Literature

**DOI:** 10.3390/jcm12113796

**Published:** 2023-05-31

**Authors:** Federico Fontana, Filippo Piacentino, Marco Curti, Anna Maria Ierardi, Andrea Coppola, Edoardo Macchi, Giuseppe De Marchi, Eliodoro Faiella, Domiziana Santucci, Lorenzo Paolo Moramarco, Filippo Del Grande, Gabriele Piffaretti, Matteo Tozzi, Andrea Imperatori, Giulio Carcano, Antonio Basile, Fabio D’Angelo, Gianpaolo Carrafiello, Massimo Venturini

**Affiliations:** 1 Diagnostic and Interventional Radiology Unit, ASST Settelaghi, 21100 Varese, Italy; 2 Department of Medicine and Surgery, Insubria University, 21100 Varese, Italy; 3 Radiology Unit, Fondazione IRCCS Ca’ Granda Ospedale Maggiore Policlinico, 20021 Milan, Italy; 4 Postgraduation School in Radiodiagnostics, Università degli Studi di Milano, Via Festa del Perdono, 7, 20122 Milan, Italy; 5 Radiology Unit, Sant’Anna Hospital, San Fermo della Battaglia, 22042 Como, Italy; 6 Department of Radiology, Campus Bio-Medico University, 00128 Rome, Italy; 7 Istituto di Imaging della Svizzera Italiana (IIMSI), Ente Ospedaliero Cantonale EOC, 6900 Lugano, Switzerland; 8 Vascular Surgery Unit, ASST Settelaghi, 21100 Varese, Italy; 9 Thoracic Surgery Unit, ASST Settelaghi, 21100 Varese, Italy; 10 General Surgery Unit, ASST Settelaghi, 21100 Varese, Italy; 11 Radiodiagnostic and Radiotherapy Unit, Department of Medical and Surgical Sciences and Advanced Technologies “GF Ingrassia”, 95123 Catania, Italy; 12 Orthopedic Surgery Unit, ASST Settelaghi, 21100 Varese, Italy

**Keywords:** pulmonary artery, aneurysm, pseudoaneurysm, endovascular, embolization, embolic agents

## Abstract

The purpose of this bicentric case series was to report the safety, efficacy, and clinical outcome of transcatheter embolization in pulmonary artery pseudoaneurysms (PAPAs). Between January 2016 and June 2021, eight patients with PAPA were subjected to transcatheter embolization. The total number of patients was eight, of which five were female, with a mean age of 62 ± 14 years (average ± standard deviation). Etiology was traumatic in 2/8 cases and iatrogenic in 6/8 cases (after positioning a Swan-Ganz catheter in 5/6 cases and a temporary pacemaker in the latter case). In a single case, the PAPA was incidentally discovered during a routine X-ray, in the remaining 7 cases, the procedure was performed in emergency settings. PAPA embolization was performed using detachable coils alone in 3 cases; coils and glue in 1 case; coils, glue, and vascular plug in 1 case; coils and non-adhesive liquid embolic agents (Onyx and Squid respectively) in 2 cases; and non-adhesive liquid embolic agent alone (Onyx) in 1 case. No peri-procedural or post-procedural complications were recorded. Both the technical and clinical success rates were 100.0%. In conclusion, endovascular embolization is a technically feasible and safe therapeutic option for patients with PAPAs.

## 1. Introduction

Pulmonary artery aneurysms (PAAs) and pulmonary artery pseudoaneurysms (PAPAs) are rare vascular lesions that involve localized dilation of a segment of the pulmonary artery [1,2]. PAAs involve all three layers of the vessel wall, while PAPAs involve only the outer layers [3]. PAPAs are associated with a high risk of rupture, and their main infectious causes include fungi, tuberculosis, bacterial pneumonia, septic emboli, and, more recently, COVID-19 [4,5,6,7,8,9,10,11,12,13,14]. Other causes of PAAs and PAPAs include vasculitis, pulmonary hypertension, lung neoplasms, and iatrogenic factors such as cardiopulmonary surgery, radiotherapy, and percutaneous procedures [15,16]. Post-traumatic PAPAs due to direct or indirect trauma and iatrogenic pseudoaneurysm formation due to Swan-Ganz catheter mispositioning or repetitive traumatic injury of permanent venous or arterial catheters can also occur [17,18,19,20,21,22,23,24,25,26,27,28,29,30,31]. The presentation of PAPAs may be asymptomatic or, often, characterized by massive hemoptysis, dyspnea, and coughing, which can quickly evolve into hypovolemic hemorrhagic shock if not treated promptly [32,33,34,35,36,37,38,39,40,41,42,43].

Unfortunately, PAPAs are often missed at initial imaging workup and not included in the differential diagnoses, leading to a high risk of mortality [44]. Conventional radiography, computed tomography, magnetic resonance, and angiography are common imaging techniques for detecting PAPAs [45,46]. While conventional radiography can detect nodular formations, it cannot differentiate them from infectious or neoplastic entities [4]. Angiography was once considered the gold standard, but computed tomography angiography (CTA) has since taken over due to its high spatial resolution, accuracy, and ability to perform multiple reconstructions [47,48,49]. MRI is also a valid alternative, especially for patients with a history of allergy to iodine contrast or in patients on dialysis [49].

PAAs can be further classified into two types: proximal (or central) and peripheral PAAs [50]. Central PAAs involve the pulmonary trunk and the main right and left pulmonary arteries, while peripheral PAAs involve intrapulmonary arteries. In 2010, Suyoung Shin et al. introduced an angiographic classification system to aid in the endovascular management of PAPAs associated with massive hemoptysis [46]. This classification system mainly focuses on the vascularity of PAPAs and distinguishes between direct supply from the pulmonary artery and supply from systemic bronchial, as well as the presence of non-bronchial collateral arteries. This system provides treatment options based on these angiographic characteristics [50,51].

In addition to surgical options, endovascular management has emerged as a viable alternative for treating PAAs and PAPAs [18]. Endovascular techniques include coil embolization, covered stent grafting, and endovascular glue injection [19,20,21]. These minimally invasive procedures have shown promising results, particularly in patients with high surgical risk or in those with recurrent disease after surgery. However, long-term outcomes are still uncertain, and careful patient selection is crucial. In conclusion, both surgical and endovascular options play an important role in the management of PAAs and PAPAs, and the choice of treatment should be tailored to the individual patient.

In the case of PAPAs, endovascular therapy is currently considered the preferred treatment option due to its minimally invasive nature, lower risk of complications, and reduced impact on pulmonary capacity.

The endovascular approach, transcatheter embolization, is usually the first method for PAAs and PAPAs management. Since the pulmonary circulation is terminal, it is not necessary to embolize the front and the back door of the aneurysm as in other districts. However, caution is required during micro-traumatic approaches with catheters and guidewires as pseudoaneurysms are fragile and have a higher risk of rupture. Transcatheter embolization is typically the first method for managing PAAs and PAPAs, using a variety of embolizing materials such as vascular plugs, coils, and adhesive and non-adhesive liquid embolic agents [12,13,14,18,31,44,45,52,53]. An alternative option is the use of a covered stent with PAA exclusion, which is reserved for the main branches of pulmonary arteries with a straight course. While there are no shared guidelines or large studies comparing the two approaches in terms of complications and therapeutic success, individualized treatment plans should be tailored to the patient’s specific needs.

Our purpose was to retrospectively report the safety, efficacy, and clinical outcome of transcatheter embolization in PAAs and PAPAs, and to compare our data with those in the current literature.

## 2. Materials and Methods

### 2.1. Study Design and Data Collection

This retrospective case series was performed in accordance with the Declaration of Helsinki and with policies approved by the Ethics Councils. All patients involved in the study signed a written informed consent form for embolization and a specific institutional procedure-related consent that covers retrospective observational studies. We performed a retrospective analysis of patients treated with an endovascular approach for PAAs and PAPAs between 2015 and 2021 at the interventional radiology units of the Circolo Hospital (Varese) and Policlinico “Ca Granda” (Milan). Age, gender, vascular lesion cause and site, diagnostic imaging technique, symptomatic vs. asymptomatic patient, elective vs. emergent procedure, and the embolic agents used were analyzed and recorded (Table 1). The rate of technical and clinical success was analyzed. Our data were compared with data in the current literature (Table 2).

### 2.2. Embolization Procedure

All PAPAs embolization procedures were performed in an angiographic room by experienced interventional radiologists (more than 10 years) using digital subtraction angiography (DSA) (Allura Xper FD20, Philips Medical Systems, Best, The Netherlands). Local anesthesia was given in the only case treated in election, while in the remaining 7 cases, the patients were sedated and intubated, therefore local anesthesia was not necessary. A diagnostic angiography through a transfemoral or transjugular approach was performed.

After the diagnostic angiography confirming aneurysm size/location, efferent/afferent vessels characteristics, and embolization feasibility, a 5 Fr vertebral catheter (Cordis, Miami, FL, USA) was placed in the main trunk of the pulmonary artery and subsequently at the origin of the involved artery to perform the angiography. Subsequently, a 2.9 French microcatheter (Terumo, Progreat, Tokyo, Japan) was used to perform the super-selective angiography. Different embolic agents were used; Detachable coils, N-butyl-2-cyanoacrylate (Glubran 2, GEM, Viareggio, Italy), Onyx (ev3 Endovascular, Inc. Plymouth, MN, USA), Squid (Squid Peri, Emboflu, Gland, Switzerland), and vascular plugs (Amplatzer Vascular Plug, Medtronic, Dublin, Ireland). The difference in embolizing materials was dictated by the period of execution (procedures performed between January 2016 and June 2021) and by the preferences of the operator. For fusiform aneurysms, the efferent vessels were usually embolized first, followed by the aneurysm sac and then the final occlusion of the afferent vessel. In the case of saccular aneurysms involving only the arterial side wall with a narrow neck and no efferent vessels, only the sac was embolized.

### 2.3. Outcome Evaluation

Technical success was considered as the exclusion of PSA at the control DSA at the end of the procedure resulting in complete embolization of the sac and the absence of reperfusion of downstream territories. Clinical success was evaluated as the stabilization of vital parameters in patients in hemorrhagic shock, interruption of hemoptysis, and absence of the need for hemodynamic support.

Peri-procedural and post-procedural complications were assessed. Clinical outcome, hospitalization time, and eventual PAPA revascularization were also evaluated. Contrast-enhanced CTs were performed 1, 6, and 12 months after the procedure.

### 2.4. Review Method

The structure of this narrative review and the search methodology have been enumerated under the following predetermined inclusion criteria: (1) Patients undergoing percutaneous embolization for the management of pulmonary pseudoaneurysm; (2) clinical outcomes, follow-up, and complications reported; (3) full-text publications available in English; (4) publication date between 2015 and 2022. A literature search was performed in September 2021 in PubMed (MEDLINE) for studies that matched the eligibility criteria using the keywords, “embolisation” or “embolization” or “pulmonary” or “pseudoaneurysm” or “aneurysm”. Every search was then conducted for every chapter of this review. An additional manual search of the bibliographies of each included study was done to identify studies not covered by the PubMed search.

## 3. Results

At our two centers, eight patients with PAPA were subjected to transcatheter embolization between January 2016 and June 2021. This cohort included three males (average age = 59 years) and five females (average age = 63.8 years). Patient characteristics, PAPA characteristics, procedure-related data, and follow-up data have been summarized as follows: Patient characteristics (sex, age, symptomatology), PAPA characteristics (etiology, location, diameter, morphology, imaging detection, imaging pre-embolization), procedure-related data (emergency vs. elective, transfemoral vs. transjugular approach, target vessel sacrifice, embolic materials, technical success, clinical success, complications, hospitalization time) and follow-up (clinical outcome, CT control) data (Table 1).

Seven of the eight patients with ruptured PAPA were treated in an emergency setting due to massive hemoptysis. In five patients, ruptured PAPAs were caused by incorrect positioning of the Swan-Ganz catheter during the intra-operative monitoring in cardiac surgery. In two patients, ruptured PAPAs occurred due to a major contusion trauma (horseback riding fall, car accident). All these patients presented important hemoptysis, cough, hypotension, and tachycardia, and the PPA was identified during a CTA performed in the emergency setting.

In a single case, the PAPA was incidentally discovered during a routine X-ray and the procedure was performed in an elective setting. After the procedure, the patient was admitted to the thoracic surgery department and then discharged after 3 days in excellent clinical condition.

In seven cases, the patients were sedated and intubated, while in the single elective case, the patient was conscious.

**Figure 1:**
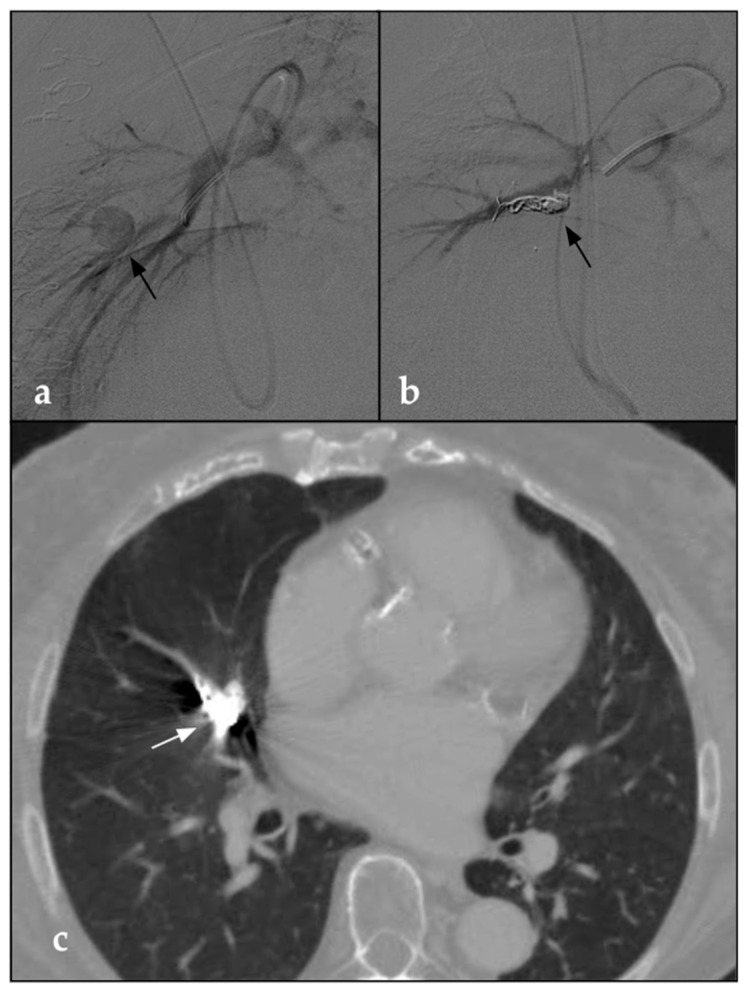
(**a**) DSA parasagittal image shows a 5 F catheter with the distal end at the level of the origin pulmonary artery tributary of the middle right lobe. The arteriographic study demonstrates a voluminous round-shaped pseudoaneurysm in the middle portion of the tributary branch of the middle right lobe (black arrow). (**b**) Post-procedural parasagittal DSA shows the complete embolization of the pseudoaneurysm through the deployment of detachable coils (black arrow). (**c**) Post-procedural CTA on the axial plane taken 1 month after the procedure confirms the complete embolization of the vascular lesion in the presence of coils (white arrow). No signs of pulmonary infarction are detectable.

**Figure 2:**
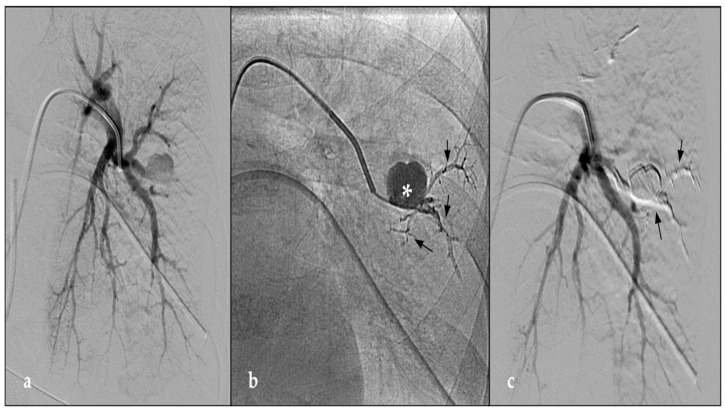
(**a**) DSA paracoronal image shows 5 F vertebral catheter (Cordis, Terumo, Tokyo, Japan) with the distal end at the level of the origin pulmonary artery tributary of the left lower lobe. The arteriographic study demonstrates a voluminous round-shaped pseudoaneurysm in the distal portion of the tributary branch of the anterior segment of the left lower lobe. (**b**) Post-procedural fluoroscopy control demonstrates the distribution of the radiopaque embolizing fluid inside the aneurysmal sac (asterisk) and efferent vessels (black arrow). (**c**) Post-procedural DSA shows the complete embolization of the pseudoaneurysm through the injection of NALEA (black arrow), with complete vascular ligation. Indeed, both inflow and outflow tracts are occluded.

The venous approach was the right jugular in six cases, and the right femoral in two cases. Six PAPA embolization procedures were performed using coils only in three cases (Figure 1); coils and glue in one case; coils, glue, and vascular plug in one case; coils and non-adhesive liquid embolic agents (Onyx and Squid, respectively) in two cases; and non-adhesive liquid embolic agent alone (Onyx) in one case (Figure 2). No peri-procedural or post-procedural complications were recorded.

The seven patients treated in emergency settings required long hospitalization in the intensive care unit (average of 5.5 days) after the procedure due to poor hemodynamic balance, the presence of clots in the bronchial tree, and the patient’s comorbidities. Patients were discharged after their general condition and breathing capacity were restored.

After the embolization, the patient treated in an elective setting was admitted to the thoracic surgery department and then discharged after 3 days in excellent clinical condition. In all patients, contrast-enhanced CT at 1, 6, and 12 months did not show signs of PAPA revascularization. Both technical and clinical success were achieved in all cases (100%), with no need for reintervention.

## 4. Review of the Literature

A total of 22 papers/case reports on PAAs/PAPAs embolization published after 2015 were collected and shown in Table 2.

## 5. Discussion

The most common causes of PAPAs and PAAs are blunt or penetrating trauma, primary or metastatic lung neoplasia, bronchiectasis, lung abscess, and other acute or chronic inflammatory lung diseases [46].

A frequent cause of PAPA is mispositioning or traumatic action of Swan-Ganz catheter: in our series, we identified PAPA formation and consequent embolization in 62.5% of the cases.

The main causes represented in our recent literature review were infective (36.7%), paraneoplastic (31.8%), Swan-Ganz-induced (13.6%), traumatic (9%), post-surgical (4.5%), and iatrogenic (4.5%) [7,8,9,10,11,12,13,14,23,24,25,26,31,32,44,54,55,56,57,58].

PAPAs are rare but often fatal and, in case of rupture, are associated with a mortality rate of about 50% [18]. The alternative scenario is accidentally discovering PAPA during a radiological examination or diagnosing it due to massive hemoptysis [40]. In asymptomatic patients, Kalra-Lall and Colleagues stated that PAPAs and PAAs are so rare that they are often not included in differential diagnoses and may be undiagnosed at the first imaging workup [43]. After diagnosis, three different approaches were followed: a wait-and-see approach, surgical intervention, or endovascular treatment. The “wait and see” approach is rarely considered due to the high risk of rupture of the aneurysmal sac [10] and is probably more suitable in the case of stable PAAs than PAPAs over time.

Nowadays, the surgical approach is generally avoided in the first instance because of the high risk of mortality; surgery is used for cases that cannot be managed with endovascular therapy or when the latter has not been effective [32]. Given the low risk of complications and the high success rate, the endovascular approach is currently the most widely used.

Endovascular treatment options in visceral and peripheral aneurysms/pseudoaneurysms generally include transcatheter embolization (widely used and almost always feasible), covered stent, flow diverting stent, bare stent-assisted coil embolization, and percutaneous approach [59]. To our knowledge, flow-diverting stent and bare stent-assisted coil embolization were not reported in the management of PAAs and PAPAs. The percutaneous approach was chosen in the case of PAPAs (or PAAs) involving pulmonary peripheral branches often superficially located using different embolizing agents such as thrombin or glue, injected under CT, ultrasound, and/or fluoroscopy guidance [9,29,60].

In our experience, embolization was preferred in all cases of PAPAs. Additionally, the presented studies also reported endovascular management as the first choice in more than 90% of cases (20/22). The endovascular approach was effective and definitive in all cases except the case described by Knaus et al., in which embolization was adopted as a “bridge therapy” to surgical treatment because the lesions were too numerous and diffusely distributed [39].

Several embolizing agents have been used with good therapeutic results: coils, vascular plugs, covered stents, thrombin, adhesive, and non-adhesive liquid embolic agents. In the literature, coils are the most widely used embolic agents, either alone or in combination with other embolizing agents [11,12,13,44,45,61]. Detachable coils, which feature better control during release with lower migration risk compared with pushable coils, are associated with an intraoperative technical success rate of 89% [41]. Aneurysmal sac revascularization was reported in almost 50% of the cases [44]. Coils may be combined with other embolic materials such as gelfoam or non-adhesive liquid embolic agents [25,44] to increase their embolizing power, hence preventing aneurysm revascularization, for example in patients undergoing anticoagulant therapy [26,31,44]. Other mechanical devices often used in PAPA or PAA embolization are vascular plugs, which have the major advantage of achieving mechanical obstruction of the proximal feeding artery [14,18,23,45,58], despite often requiring the use of large sheath introducers. In addition, the distensibility of the pulmonary arteries necessitates an oversize for the correct choice of vascular plug size. If the main target is to embolize the proximal feeding artery, covered stents may be a viable alternative, as they can preserve distal blood flow by excluding vascular lesions [24,55]. However, covered stents are associated with a higher risk of occlusion, migration, and infection, and require large sheath introducers.

Regarding the choice of stent size, the possibility of using balloon expandable stents allows correct sizing, thus avoiding the problem of distensibility of pulmonary arterial vessels; however, they are poorly documented in the literature [24].

Another category of embolization material is liquid agents, including thrombin, adhesive embolic agent (AEA), and non-adhesive embolic agent (NALEA). Thrombin, a non-radiopaque embolization liquid, plays a key role in the blood coagulation cascade; this liquid agent is often used for percutaneous embolization of peripheral pseudoaneurysms using an ultrasound-guided approach. To our knowledge, no cases of PPA embolization using thrombin have been described, probably due to poor controllability under fluoroscopy [62]. N-butyl-2-cyanoacrylate (Glubran 2, GEM) is a fast-hardening AEA characterized by a high embolizing power by permanently occluding the vessel. This liquid is characterized by low viscosity and can be injected into small, tortuous arteries via microcatheter. Glubran is a non-radiopaque adhesives liquid, which makes it impractical to handle unless mixed with a contrast agent, typically LipiodolUltra Fluid (Guerbet, Paris, France, EU), an iodinated contrast agent that provides radiopacity to the compound. The polymerization speed of Glubran is also modified by mixing and depends on the concentration of Lipiodol; the higher the concentration of Lipiodol, the longer the polymerization time. In the literature, Glubran is often used during PPA embolization in emergency settings, either alone or in combination with other embolization materials, due to its high embolizing power [8,9,26,31,45]. Among the main disadvantages of Glubran are the difficulty in handling and the high risk of non-target embolization during catheter removal and catheter flushing [18].

Another category of liquid embolizing agents is the NALEA, of which Onyx (ev3 Endovascular, Inc. Plymouth, MN, USA), Squid (Squid Peri, Emboflu, Gland, Switzerland), PHIL (MicroVention, Tustin, CA, USA), and Easyx (MicroVention, Tustin, CA, USA) are all included [44,61]. NALEAs have gained popularity because of their ‘magma-like’ flow, allowing good control of the embolic material. They can also be used alone or combined with other occlusive agents to enhance their embolic power [44,44]. NALEAs have multiple advantages over adhesive agents: excellent embolizing capacity, can reach vessels with a diameter of 80 microns with a low tendency to induce off-target embolization, and have reduced risk of microcatheter entrapment. In addition, the magma effect permits significant intravascular migration of the embolic fluid before it solidifies, resulting in complete embolization of the vascular lesion on both the inflow and outflow sides, thereby achieving complete vascular ligation and reducing the risk of distal revascularization.

NALEAs get their radiopacity from the tantalum (Onyx, Squid) or iodine (Phil, Easyx) in their formulation [61].

Their composition and the homogeneous distribution of radiopaque compounds results in a significant reduction of beam hardening artifacts compared with those generated by mechanical embolization devices (coils, vascular plugs, and stents). These CT artifacts can cause severe limitations in the discrimination of the efficacy of the embolization procedure, preventing, in some cases, the exclusion of revascularization of the PPA with certainty.

The use of Squid in association with a CE-CT reconstructed by the SEMAR soft-ware (single-energy iterative retrospective metal artifact reduction software that reduces near-field artifacts generated by both high- and low-density implants, Aquilion, PRIME SP Software version V8.4, Canon Medical, Rome, Italy) has allowed the minimization of beam hardening artifacts and provided the certainty of PPA ex-clusion during postoperative CT follow-ups [44].

The main disadvantages of NALEA include the need to use compatible microcatheters, the cost, and the mandatory use of dimethyl sulfoxide (DMSO). Indeed, NALEA needs a previous injection of DMSO to allow conduction within the microcatheter and to maintain the fluid formula; however, this solvent can cause vasospasm, damage to the endothelial wall, and pain. Of the various embolization materials that can be used in the case of embolization of a PPA, only microparticles are not recommended. Indeed, the presence of a vascular shunt between the pulmonary arterial and venous circulation cannot always be excluded, which would allow the microparticles to migrate to the systemic circulation and cause dangerous intracranial and cardiac embolization.

In our review of the literature as well as in our experience, we found that there is no uniformity in the choice of embolic material for embolization. However, we confirmed that controlled-release coils are the most widely used embolic agents, have very good efficacy values, and are also the most widely used in combination with other embolic materials.

Concerning the number of patients and causes, our case series is, to our knowledge, the largest and most heterogeneous in the literature (eight patients). Indeed, the majority of the analyzed studies are mainly case reports with either one or (a maximum of) two patients. The only study describing a case series (five patients) was presented by Torikai et al.; however, the only etiological cause reported in that study was infectious [56].

In our case history, we used NALEA in two cases, with excellent results and without any complications such as pulmonary infarctions or lung abscesses.

Chronologically, the two cases treated with NALEA are the most recent, as our experience with this type of embolic fluid has greatly increased in recent years, hence we were able to achieve higher confidence during its injection.

To summarize, based on our personal experience, NALEAs offer very good results, either alone or in combination, and are characterized by a low risk of revascularization of the sac. Furthermore, their embolizing power is not dependent on the coagulation status of the patient.

## 6. Study Limitations

This study had some limitations: (1) The number of patients studied is small; however, it is still the largest study in the literature. (2) Only two centers are included. (3) Case series are characterized by significant patient selection bias; moreover, both urgent and elective cases were included. (4) Different embolic materials were used, with the criterion for choosing the material being the interventional radiologist’s preference. Thus, true comparisons could not be provided. (5) The techniques presented did not include percutaneous puncture. (6) The Literature review is not systematic, which makes it less precise and accurate.

## 7. Conclusions

In our experience, several embolic agents were successfully used for the treatment of PAPA—either alone or in combination with other agents—for example, NALEAs, with high technical and clinical success and low re-intervention rates in patients considered as high risk for open repair. In conclusion, several embolic materials may be used successfully for endovascular embolization in patients with PAPA. Due to limited data, further cases are needed to validate these outcomes.

## Figures and Tables

**Table 1:** Patient, PAPA characteristics; procedure-related, follow-up data.

	Patient Characteristics		PAPA Characteristics	Procedure-Related Data				Follow-Up Data
Pt #	Sex	Age (y.o.)	Etiology	Location	Detection	Diagnosis	Embolic Material	CTA ad 12 Months
**1**	M	73	Swan-Ganz	Right Lower lobe	CTA	CTA	Coils and Glue	No PAPA revascularization
**2**	F	35	Fall from horse	Left Lower lobe	CTA	CTA	Coils	No PAPA revascularization
**3**	F	65	Swan-Ganz	Right Medium lobe	CTA	CTA	Vascular plug and Glue	No PAPA revascularization
**4**	F	72	Swan-Ganz	Right Lower lobe	CTA	CTA	Coils	No PAPA revascularization
**5**	F	75	Swan-Ganz	Right Medium lobe	CTA	CTA	Coils	No PAPA revascularization
**6**	M	56	Car accident penetrating trauma	Right Lower lobe	CTA	CTA	Coils and AEA	No PAPA revascularization
**7**	M	48	Temporary pacemaker	Left Lower lobe	Thoracic X-ray	CTA	Coils and EVOH liquid	No PAPA revascularization
**8**	F	72	Swan-Ganz	Left Lower lobe	CTA	CTA	EVOH liquid	No PAPA revascularization

**Table 2:** Case reports published from 2015 to 2021 were analyzed. The table presents publications on the embolization of pulmonary pseudoaneurysms (PAPAs) in the last 7 years. Column 4 shows that almost all are case reports—except for rows 3, 5, 7, and 17—although only Torikai (line 7) has a substantial number of cases with this rare condition. Column 5 reports the causes described in the literature, with an infectious cause prevalence of about 30% (in agreement with the literature), but in disagreement with our case series, which did not find any infectious causes. In column 6, we present a distribution of pseudoaneurysms in line with the literature, with most cases occurring in the right lung. Column 7 reports the embolizing materials, demonstrating that there is no ideal agent, but rather that this depends largely on the experience of the interventional radiologist.

	Author	Journal	Year	n° of Cases	Cause	Location	Embolic Agents
1	Ierardi	BMJ Case Rep	2015	1	Swan-Ganz	Middle Lobe	Glue and Vascular Plug
2	Petersen	BMJ Case Rep	2015	1	Swan-Ganz	Middle lobe	Coils
3	Chatterjee	Respir Med Case Rep	2015	3 (same patient)	Rasmussen Pseudoaneurysm (Tuberculosis)	Left upper lobe	Glue
4	Garg	Clin Case Rep.	2015	1	Lung metastasis of synovial sarcoma	Left main pulmonary artery	Covered stent
5	Rudziński	Postepy Kardiol Interwencyjnej	2016	2 (2 patients)	Swan-Ganz	Right lower lobe	1 case treated with coils, 1 with vascular plug
6	Pedicini	J Vasc Interv Radiol	2017	1	Mitral valve replacement	Middle lobe	Glue and Coils
7	Torikai	J Vasc Interv Radiol	2017	7 PPA (5 patients)	3 Inactive Tuberculosis, 1 Cavitary infection, and 1 nontuberculous mycobacteriosis	3 Left upper lobe, 2 Right upper lobe	Glue
8	Urlings	J Vasc Intev Radiol	2017	1	Lung cancer	Left lower lobe	Fluoroscopic and ultrasound-guided embolization with coils and Glue
9	Shnayderman	Radiol Case Rep	2017	1	Penetrating thoracic trauma	Left lower lobe	Coils and vascular plug
10	Ishimoto	Ann Thorac Cardiovasc Surg	2018	1	Mycotic infection during chemotherapy for acute myeloid leukemia	Right Lower Lobe	Lobectomy
11	Gabrielli	Ann Thorac Surg	2018	1	Septic emboli from bacterial endocarditis	Right lower lobe	Coils
12	Lazic	BMJ Case Rep	2019	1	Bacterial pneumonia	Right upper lobe	Wait and see
13	Koirala	Cureus	2020	1	Lung carcinoma over-infected by Klebsiella	Right lung (bronchial artery)	Glue e coils
14	Piacentino	Acta Biomed	2021	1	Temporary pacemaker	Left lower lobe	NALEA and coils
15	Jajodia	Rad case reports	2021	1	COVID	Right lower lobe	Coils
16	Tanaka	Respir Med Case Rep	2021	1	Lung abscess	Middle lobe	Coils
17	Vaid	Clin Med (Lond)	2021	2 (same patients)	Metastasis from uterine leyomiosarcoma	Right lower lobe	Vascular plugs
18	Grange	Rad case reports	2021	1	Tuberculosis	Left lower lobe	Percutaneous Glue embolization
19	Khurram	Eur J Radiol Open	2021	1	COVID	Left upper lobe	Vascular plug
20	Doyle	BMJ Case Rep	2021	1	Previous lobectomy and radiotherapy for lung sarcoma (7 years before)	Right upper lobe	Covered stent
21	Lutz	Rad case reports	2021	1	Previous lobectomy and radiotherapy for lung adenocarcinoma (7 years before)	Middle lobe	Coils (first attempt) NALEA (second attempt)
22	Knaus	Ann Thorac Surg	2021	Multiple (same patient)	Gunshot	Right lung	Surgical and coil embolization (1–2 attempts) leading to Right pneumonectomy

## Data Availability

The data presented in this study are available in the article (Table 1 and Table 2).
